# Plant Mediated Green Synthesis of CuO Nanoparticles: Comparison of Toxicity of Engineered and Plant Mediated CuO Nanoparticles towards *Daphnia magna*

**DOI:** 10.3390/nano6110205

**Published:** 2016-11-09

**Authors:** Sadia Saif, Arifa Tahir, Tayyaba Asim, Yongsheng Chen

**Affiliations:** 1 School of Civil and Environmental Engineering, Georgia Institute of Technology, Atlanta, GA 30332, USA; 2 Department of Environmental Science, Lahore College for Women University, Lahore 54000, Pakistan; arifa.tahir@gmail.com (A.T.); tayyabasim2008@gmail.com (T.A.); 3 Department of Biochemistry, Quetta Institute of Medical Science, Quetta 87300, Pakistan

**Keywords:** copper oxide (CuO) nanoparticles, green synthesis, *Pterospermum acerifolium*, bio toxicity, *Daphnia magna*

## Abstract

Research on green production methods for metal oxide nanoparticles (NPs) is growing, with the objective to overcome the potential hazards of these chemicals for a safer environment. In this study, facile, ecofriendly synthesis of copper oxide (CuO) nanoparticles was successfully achieved using aqueous extract of *Pterospermum acerifolium* leaves. *P. acerifolium*-fabricated CuO nanoparticles were further characterized by UV-Visible spectroscopy, field emission scanning electron microscopy (FE-SEM), energy dispersive X-ray (EDX), Fourier transform infrared spectroscopy (FTIR), X-ray photoelectron spectroscopy (XPS) and dynamic light scattering (DLS). Plant-mediated CuO nanoparticles were found to be oval shaped and well dispersed in suspension. XPS confirmed the elemental composition of *P. acerifolium*-mediated copper nanoparticles as comprised purely of copper and oxygen. DLS measurements and ion release profile showed that *P. acerifolium*-mediated copper nanoparticles were more stable than the engineered CuO NPs. Copper oxide nanoparticles are used in many applications; therefore, their potential toxicity cannot be ignored. A comparative study was performed to investigate the bio-toxic impacts of plant-synthesized and engineered CuO nanoparticles on water flea *Daphnia.* Experiments were conducted to investigate the 48-h acute toxicity of engineered CuO NPs and plant-synthesized nanoparticles. Lower EC_50_ value 0.102 ± 0.019 mg/L was observed for engineered CuO NPs, while 0.69 ± 0.226 mg/L was observed for plant-synthesized CuO NPs. Additionally, ion release from CuO nanoparticles and 48-h accumulation of these nano CuOs in daphnids were also calculated. Our findings thus suggest that the contribution of released ions from nanoparticles and particles/ions accumulation in *Daphnia* needs to be interpreted with care.

## 1. Introduction

Green chemistry focuses on the production of desired products without generation of hazardous intermediate byproducts in chemical reaction processes. Integrating green chemistry principles into nanotechnology has led to the identification of environmentally friendly reagents that are multifunctional, in that they can serve as a reducing agent as well as a capping agent [1,2]. Synthesis of nanoparticles (NPs) can be performed using a number of routinely used chemical and physical methods [3,4,5,6,7]. Plants and their related materials for production of nanomaterials are not only ecofriendly alternatives, but they are also cost effective. Synthesis of copper oxide (CuO) nanoparticles has been performed using extracts of soybeans [8], gum karaya [9], bark extract [10], leaf extract [11,12], fruit [13,14], tea and coffee powder [15], peel extract [16] and flower extract [17].

Development in large-scale production for both metallic and nonmetallic nanoparticles has introduced risk to the environment and human health [18,19]. Improper disposal of nanomaterial waste by labs as well as industry is an alarming threat to the ecosystem as well as aquatic life. With this perspective, researchers are focusing on the green synthesis of nanomaterials [1]. The aim is to protect the environment and human health from toxic impacts of nanomaterials and their derived complex compounds and at the same time safely utilize nanomaterials. Environmental and biological risks for copper nanoparticles have been investigated by many researchers. However, the toxicity of nanosized and bulk CuO metal oxide nanoparticles to bacteria [20], yeast [21], green algae [22], crustaceans *Daphnia magna* and *Thamnocephalus platyurus* [23] and isopods [24] are well documented. Researchers have also found toxic and inhibiting growth impacts to terrestrial plants caused by CuO metal oxide nanoparticles [25,26]. The lethal effect of CuO nanoparticles on aquatic organisms has been verified by several studies, which include zebrafish (*Danio rerio*) [27,28], sea anemones (*Exaiptasia pallida*) [29], fresh water shredders [30], blue mussels (*Mytilus edulis*) [31] and goldfish [32]. Cytotoxicity and genotoxicity of CuO nanoparticles have been investigated in humans as well as in animals. Finally, CuO nanoparticles have been determined more toxic to human cells with their ability to damage DNA than bulk micrometer particles [33,34].

**Figure 1:**
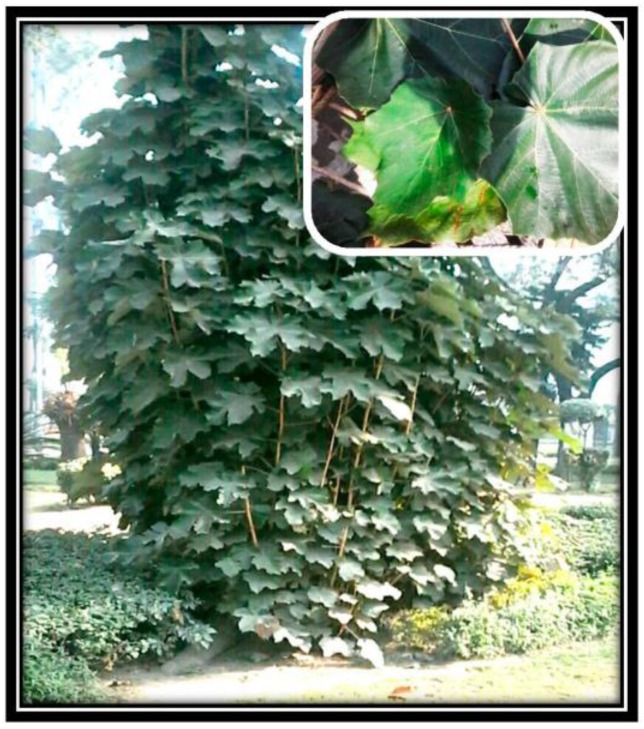
*Pterospermum acerifolium* tree.

In this study, we synthesized nanoparticles by leaf extract of *Pterospermum acerifolium*, which is an angiosperm belonging to the family Malavacea distributed in Southeast Asia. The common name is “Kanack champa” in Pakistan (Figure 1). The bark and leaves have been used for pharmaceutical purposes [35]. Facile synthesis of CuO nanoparticles was performed without using any extensive chemicals or excess energy. Further CuO NPs were characterized by field emission scanning electron microscopy (FE-SEM), Fourier transform infrared spectroscopy (FTIR), X-ray photoelectron spectroscopy (XPS) and dynamic light scattering (DLS) for zeta potential and hydrodynamic size measurements. The study revealed the toxicological impacts of plant-synthesized CuO nanoparticles on *Daphnia magna* in comparison with engineered nanoparticles. To the best of our knowledge, this is the first study to evaluate the acute toxicity of plant-synthesized copper nanoparticles to model *Daphnia magna*. The 48-h acute toxicity tests were performed to calculate the effective concentration (EC_50_) value for both engineered and plant CuO nanoparticles to *D. magna*. Moreover, ion release, accumulation of nanoparticles in *Daphnia* and the relative contribution of nanoparticles and ions to accumulation was investigated for both engineered and plant CuO nanoparticles.

## 2. Materials and Methods

### 2.1. Materials and Chemicals

Tree leaves of *Pterospermum acerifolium* were collected from Bagh-e-Jinnah Lahore, Pakistan. Copper(II) nitrate trihydrate Cu(NO_3_)_2_·3H_2_O and nitric acid (69% HNO_3_) was purchased from Sigma Aldrich (St. Louis, MO, USA). Cu metal standard (1000 µg/mL in 2% HNO_3_) for inductively coupled plasma optical emission spectrometry (ICP-OES) analysis was purchased from High-Purity Standards, USA. For the toxicity bioassay, test organism *D. magna* were obtained from the Carolina Biological Supply Company (Burlington, NC, USA) and cultured in Daniel Lab, Georgia Institute of Technology. The culture medium was renewed two times each week, and the *Daphnia* were fed daily with food purchased from the same company. The culture was maintained at a constant temperature (22 ± 2 °C) with a natural light-dark cycle.

### 2.2. Synthesis of Plant CuO Nanoparticles

CuO nanoparticles were synthesized by aqueous extract of *P. acerifolium*. Extract was prepared by the maceration method using 20 g of fine leaf powder with deionized water. Before synthesis of nanoparticles, extract was carefully filtered by making sure no solid particles were in the filter. A stoichiometric amount of copper Cu(NO_3_)_2_·3H_2_O was added in 10 mL of extract and then mixed thoroughly by a magnetic stirrer at 45–50 °C. The deep blue color of the copper salt solution changed to dark green. The mixture was placed in a preheated furnace at 400 °C for 5 min. The black fine product was obtained and stored in an airtight glass container for further use.

### 2.3. Characterization

The UV-Visible absorption spectra were recorded by the UV-Visible spectrophotometer (UV-1800; Shimadzu, Tokyo, Japan). The morphology and size of CuO nanoparticles were characterized by cold field emission scanning electron microscopy (CFE-SEM, SU8230, Hitachi, Tokyo, Japan). The Thermo Scientific™ K-Alpha^+^™ X-ray Photoelectron Spectrometer (XPS) System (Thermo Fisher Scientific, Waltham, MA, USA) was used to study the near-surface elemental composition of plant-synthesized CuO nanoparticles. Chemical binding of synthesized nanoparticles were analyzed using a Fourier transform infrared (FTIR) spectrometer (Perkin Elmer, Spectrum 400, Walthman, MA, USA) equipped with an attenuated total reflection (ATR) attachment. For the determination of size/size distribution and zeta potential, we used the dynamic light scattering (DLS) Nano-ZS instrument (Malvern Instruments Ltd., Malvern, UK).

### 2.4. Ion Release Profile of CuO Nanoparticles

Cu ion release from engineered and plant CuO nanoparticles at different concentrations of 1 mg/L, 10 mg/L, 100 mg/L was measured. Concentrations were prepared in Mili-Q water. Samples were taken at intervals of 1 h, 24 h, 48 h, 72 h; then, the supernatants were filtered through a syringe filter with 0.2 μm pore diameter and subjected to copper analysis by inductively coupled plasma optical emission spectrometry (ICP-OES) after digestion in 69% HNO_3_.

### 2.5. Acute Toxicity Experiments

#### 2.5.1. Bioassay

Acute toxicity tests of copper oxide nanoparticles were conducted based on the modified OECD standard procedure [36]. Test materials were engineered CuO NPs with a nominal size of 50 nm purchased from Sigma-Aldrich (Louis, MO, USA) and plant-synthesized NPs. Stock nanoparticle suspensions were prepared (30 min sonication in a water bath sonicator). Several test suspension samples with concentrations ranging from 10 to 0.05 mg/L were prepared and control was also set for biotoxicity assay. For each suspension sample with a specified concentration, 10 *D. magna* neonates from a cultured tank were placed in a 50 mL glass beaker containing 30 mL test solution. Each concentration was tested with three replicates. The test solution was renewed after 24 h to maintain the same dose of exposure. *D. magna* were not fed during the testing period. After exposure to both NPs suspensions (%), mortality was observed after 24 and 48 h.

#### 2.5.2. Assessment of CuO Nanoparticles Accumulation in *Daphnia*

Based on the EC_50_ toxicity test, two doses were selected and tested for study of accumulation of copper oxide nanoparticles in *Daphnia*. Test concentrations for plant CuO NPs were 0.1 mg/L and 0.05 mg/L and for engineered CuO NPs these concentrations were 0.05 mg/L and 0.01 mg/L. The 48 h exposure period was selected in the accumulation test and adult *Daphnia* were used. Similar to the acute toxicity tests, the accumulation experiments were conducted under a 16:8 h light/dark photoperiod (20 ± 1 °C) without feeding during the 48 h exposure period. After the 48 h exposure period, 10 mobile *Daphnia* were sampled from each exposure medium, and they then were transferred to Milli-Q water for 1–3 min. Subsequently, they were rinsed three times with fresh Milli-Q water. After rinsing, they were dried at 80 °C overnight in a preweighed glass dish before weighing on a microbalance and then digested in 69% HNO_3_ at 80 °C overnight. The Cu concentrations in the digested samples were subsequently determined by ICP-OES. Samples were analyzed from test media after 24 h exposure times to investigate the relative contribution of released ions and particles in the accumulation process. 

### 2.6. Data Analysis

Data were analyzed using Origin Pro 7.5 SRO software (Origin Lab Corporation, Northampton, MA, USA) and Microsoft Excel 2010 (Redmond, WA, USA).

## 3. Results and Discussion

### 3.1. Physicochemical Characterization of CuO Nanoparticles

**Figure 2:**
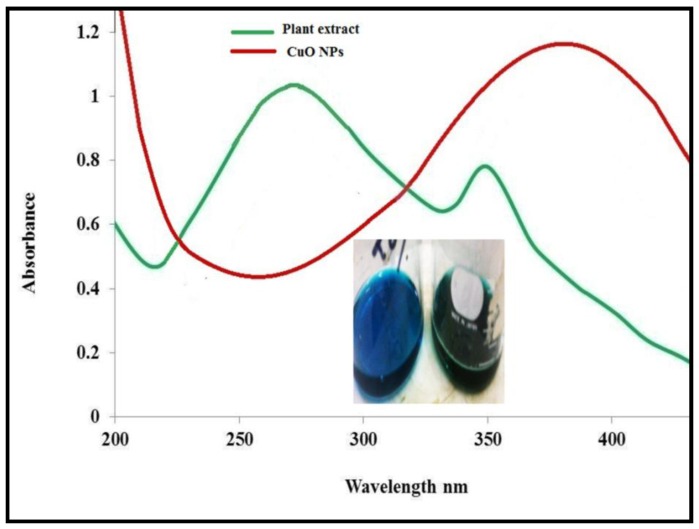
UV-Visible spectroscopy of *P. acerifolium*-synthesized copper oxide (CuO) nanoparticles (NPs).

In this study, copper oxide nanoparticles were successfully synthesized by greener approach using leaf extract of *P. acerifolium*. The formation of CuO nanoparticles was confirmed by UV-Visible spectra analysis. Figure 2 shows the absorption spectra of *P. acerifolium* leaf extract and *P. acerifolium*-synthesized CuO nanoparticles. Broad absorption peak of about 270 nm was observed for leaf extract while narrow and broad peaks at 274 nm and 383 nm were obtained from CuO nanoparticles. The results were in agreement with study of Udayabhanu et al. [37].

**Figure 3:**
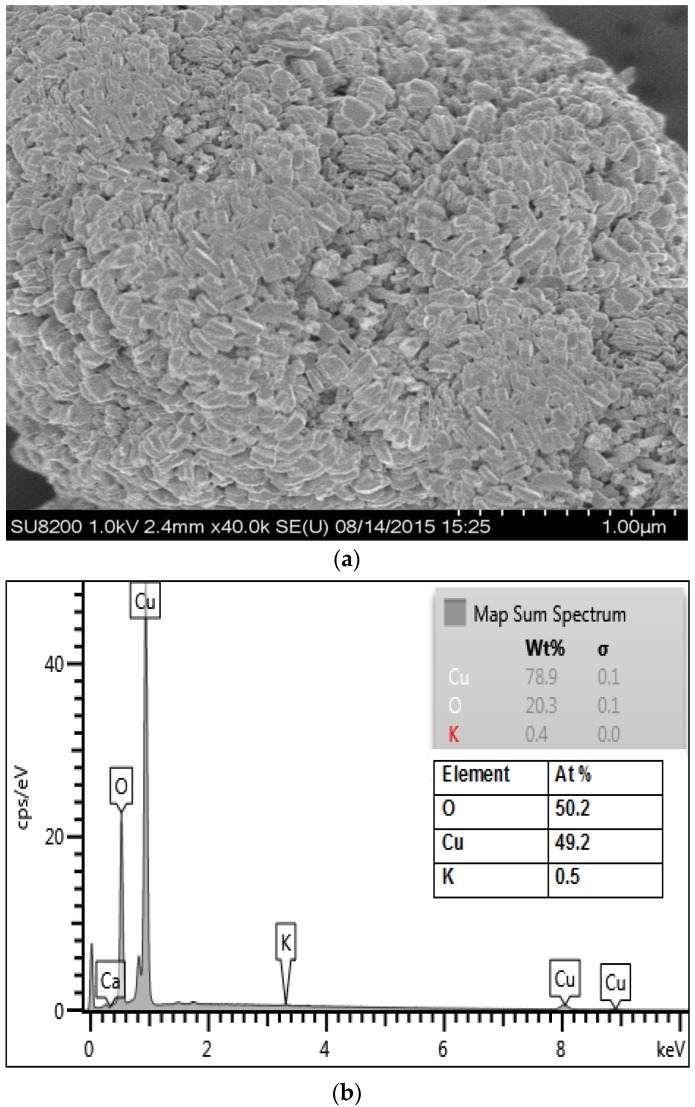
(**a**) Field emission scanning electron microscopy (FE-SEM) images and (**b**) energy dispersive X-ray (EDX) profile of plant-synthesized CuO nanoparticles.

SEM image of *P. acerifolium* (Kanak champa tree)-synthesized CuO NPs is presented in Figure 3a. Plant-synthesized nanoparticles were found to be slightly oval shaped. SEM images revealed that *P. acerifolium* CuO nanoparticles have a diameter ranging from 0.1 to 0.25 µm. Figure 3b shows the EDX profile of *P. acerifolium* CuO nanoparticles, which confirmed the signal characteristic of copper and oxygen only.

**Figure 4:**
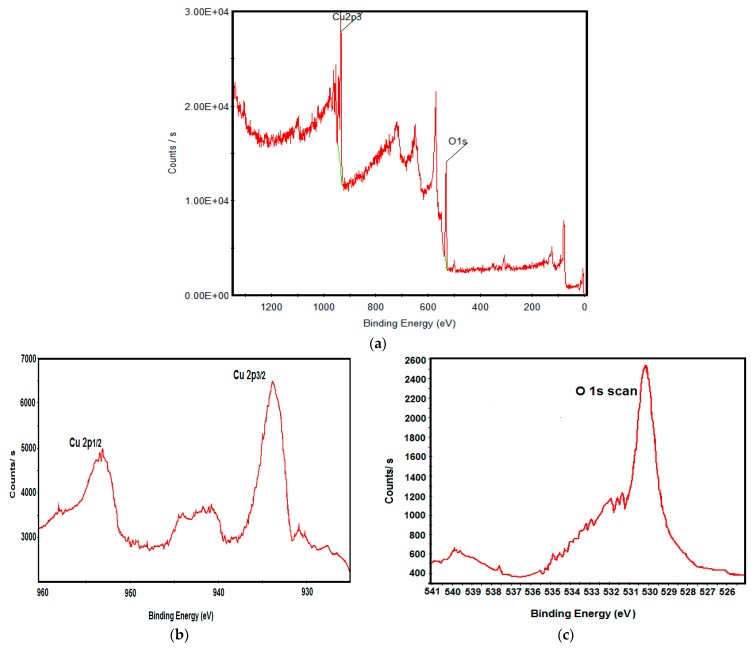
(**a**) X-ray photoelectron spectroscopy (XPS) survey spectrum of *P. acerifolium*-synthesized CuO nanoparticles; (**b**) Cu 2p scan; (**c**) O 1s scan.

For the confirmation of elemental composition of *P. acerifolium*-synthesized CuO nanoparticles, XPS analysis was performed (Figure 4). The survey XPS spectrum shows that binding energy around 934.4 eV and 952.4 eV correspond to Cu 2p_3/2_ and Cu 2p_1/2_. The intense peak at 529.8 eV corresponds to oxygen atom bond to Cu, forming CuO nanoparticles. These results are in agreement with reported data of Tamuly et al. [16]. Results proved that CuO nanoparticles were comprised of Cu^2+^ and O, having no impurities.

**Figure 5:**
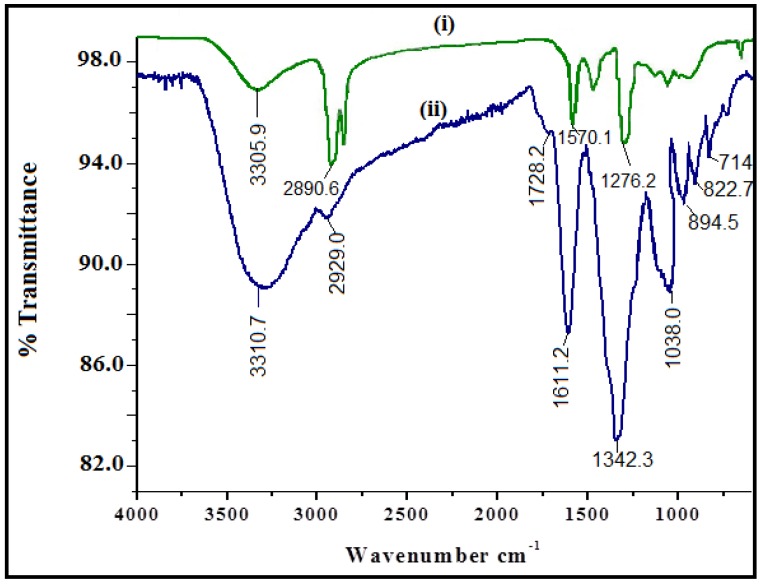
Fourier transform infrared spectroscopy (FTIR) spectra of (**i**) *P. acerifolium* leaf extract; (**ii**) *P. acerifolium*-synthesized CuO nanoparticles.

FTIR spectroscopy was employed to characterize and identify the biomolecules of leaf extract of *P. acerifolium*. FTIR spectra of *P. acerifolium* leaf extract and synthesized CuO are shown in Figure 5. Figure 5 clearly indicates the peak shifts after reaction of copper nitrate with leaf extract. The FTIR spectrum of plant-mediated CuO nanoparticle shows that the broad absorption band at 3310.7 cm^−1^ corresponds to the hydroxyl (OH) functional group in alcohols and phenolic compounds. The sharp absorption peaks were observed in the range of 1700–1000 cm^−1^. The IR band around 1611.2 cm^−1^ can be assigned to aromatic bending of alkene group (C=C). Sharp peak at 1342.3 cm^−1^ is attributed to the deformation vibration of the C–H band of alkane (CH_3_ and CH_2_) group. Absorption peak at 1038.0 cm^−1^ stretching vibration of C–O group of primary and secondary alcohols (C–O), while smaller peaks at 900–700 cm^−1^ were also assigned to the aromatic bending vibration of C–H group.

**Figure 6:**
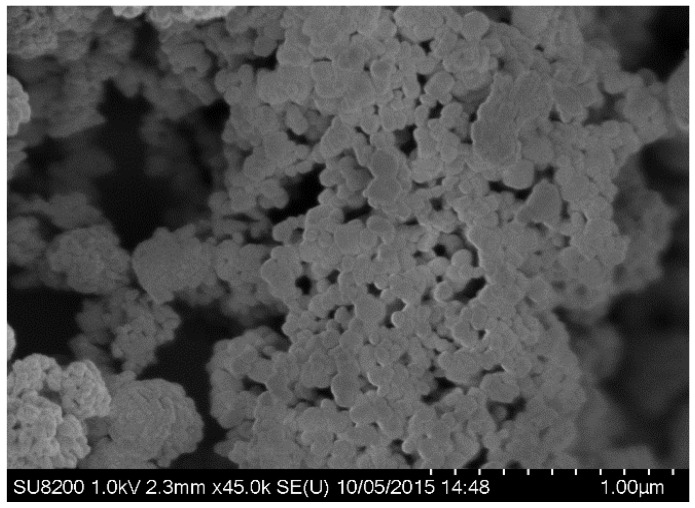
Scanning electron microscopy (SEM) image of engineered CuO nanoparticles.

**Figure 7:**
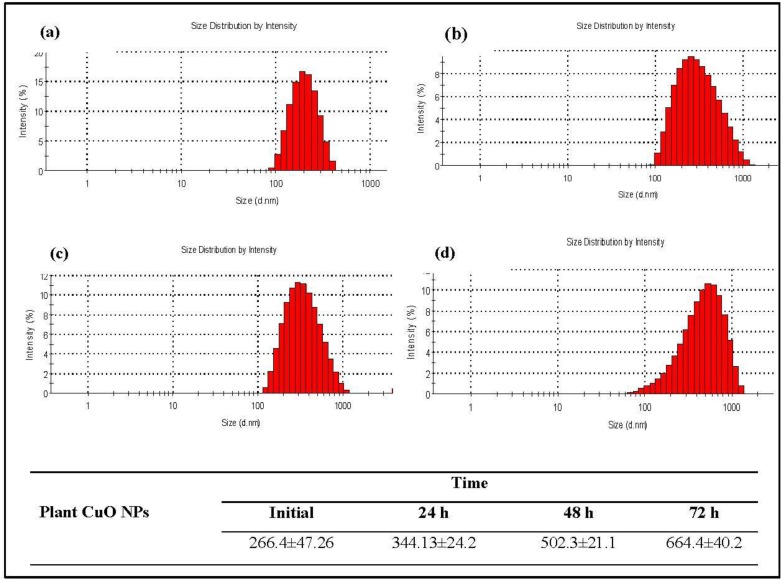
Size and size distribution of plant-synthesized nanoparticles (**a**) initial; (**b**) 24 h; (**c**) 48 h and (**d**) 72 h.

**Figure 8:**
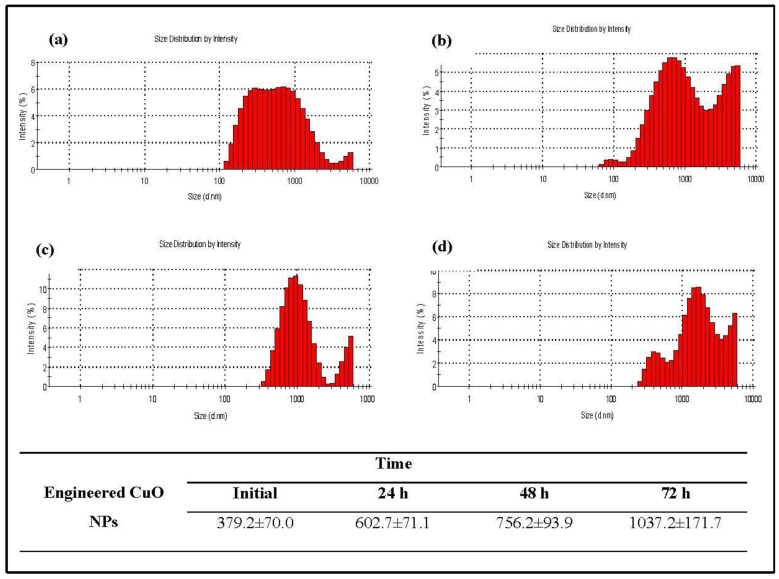
Size and size distribution of engineered copper nanoparticles (**a**) initial; (**b**) 24 h; (**c**) 48 h and (**d**) 72 h.

Dynamic light scattering (DLS) was performed to determine the hydrodynamic size (diameter) and zeta potential for both plant-synthesized and engineered CuO nanoparticles. However the engineered nanoparticles used in this study had a diameter of less than <50 nm (purchased from sigma Aldrich) (Figure 6). Data on size and size distributions of plant-synthesized and engineered CuO nanoparticles at different time intervals are given in Figure 7 and Figure 8. The particle size of plant CuO NPs shifted from 212.6 ± 47.26 nm to 634.4 ± 40 .2 nm after 72 h, while the particle size of engineered CuO NPs was found to increase from 379.2 ± 70.0 nm to 1037.2 ± 171.7nm after 72 h (Figure 8). Results revealed that the hydrodynamic size increased after a time but plant synthesis was less susceptible. 

Similarly, the zeta potential values of the two nanoparticle suspensions at different time intervals are given in Table 1. The zeta potential of the engineered CuO NPs increased from −11.7 ± 2.52 to 18.13 ± 0.60 and the plant CuO NPs shifted from −9.27 ± 1.10 to 16.25 ± 0.36 after 72-h. The zeta potential study also demonstrated that plant-synthesized nanoparticles were more stable than the engineered nanoparticles, it might be due to capping of nanoparticles by the biomolecule of *P. acerifolium.* The plant-synthesized nanoparticles showed more stability and less aggregation over the time than the engineered CuO nanoparticles when measured by zeta potential.

### 3.2. Ion Release from CuO Nanoparticles

**Figure 9:**
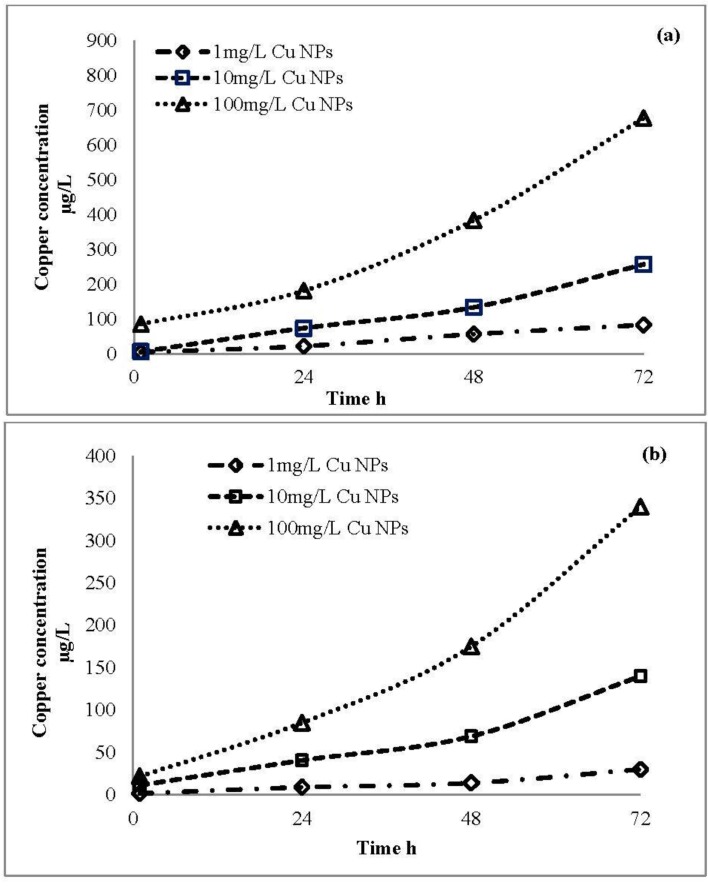
Copper ion release (**a**) engineered CuO nanoparticles and (**b**) plant-synthesized CuO nanoparticles (Mean ± standard deviation (SD) (*n* = 3)).

The profile of ion release from engineered and plant CuO nanoparticles is shown in Figure 9a,b. Test solutions with different concentrations (1 mg/L, 10 mg/L, and 100 mg/L) were tested for both engineered and plant CuO nanoparticles. Results showed that the higher the concentration of nanoparticles, the greater the ion release, and ion release increased after 72 h. From engineered CuO nanoparticles at 1 mg/L concentration in test solution, the release of copper ions was 4.3 ± 0.02 µg/L, 21.7 ± 0.01 µg/L, 56.3 ± 0.02 µg/L and 83 ± 0.01 µg/L after 1 h, 24 h, 48 h and 72 h, respectively. Test solution with 10 mg/L concentration released copper ions 5.9 ± 0.03 µg/L, 73.6 ± 0.01 µg/L, 133.3 ± 0.01 µg/L and 256.6 ± 0.05 µg/L after 1 h, 24 h, 48 h and 72 h, while 100 mg/L concentration nanoparticle solution released 85.0 ± 0.1 µg/L, 181.3 ± 0.04 µg/L, 383.3 ± 0.06 µg/L and 676.6 ± 0.09 µg/L after 1 h, 24 h, 48 h and 72 h, respectively. 

For the plant-synthesized CuO nanoparticles at test solution concentration of 1 mg/L, the ion release was 1.4 ± 0.02 µg/L, 8.9 ± 0.0 µg/L, 13.9 ± 0.05 µg/L and 30 ± 0.02 µg/L after 1 h, 24 h, 8 h and 72 h, respectively. For 10 mg/L concentration, the ion release was 11.3 ± 0.04 µg/L, 40.6 ± 0.02 µg/L, 69 ± 0.06 µg/L and 140 ± 0.04 µg/L after 1 h, 24 h, 8 h and 72 h, and for 100 mg/L, the ion release was 21.8 ± 0.07 µg/L, 85 ± 0.06 µg/L, 175 ± 0.1 µg/L and 340 ± 0.05 µg/L after 1 h, 24 h, 8 h and 72 h, respectively. Our record of CuO nanoparticles dissolution in the test medium agreed with other studies [38,39]. The CuO nanoparticles aggregate, dissolute and transform, and are affected by aquatic chemistry. The dissolution of nanoparticles also depends on factors such as the exposure concentration, chemical composition and temperature, etc. [19,40]. Findings of the study showed that the process of ion release is higher in engineered CuO than the plant-synthesized nanoparticles, and that ion release depends on concentration of the nanoparticles solution. However, the plant-synthesized CuO nanoparticles were found to be more stable; it is thought that biomolecules in plant extract which involved in synthesis act as capping and stabilizing agent.

### 3.3. Toxicity Bioassay

#### 3.3.1. Forty-Eight Hour Acute Toxicity of CuO Nanoparticles

**Figure 10:**
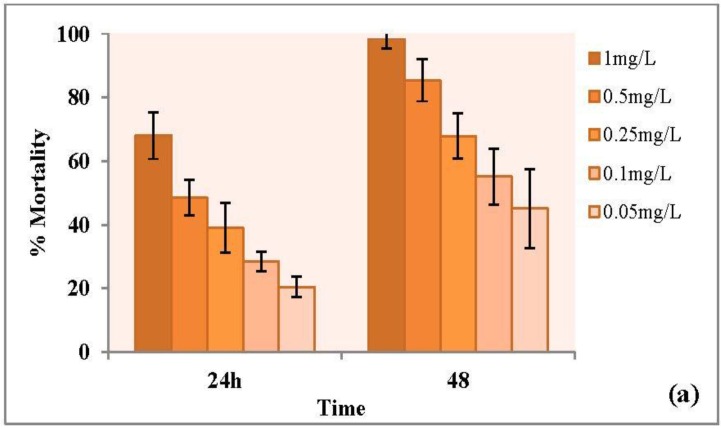

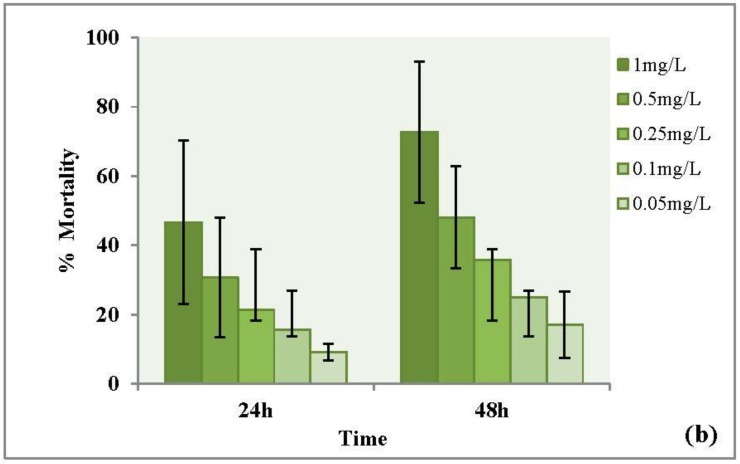
Acute toxicity by nanoparticles (**a**) engineered CuO and (**b**) plant-synthesized CuO (Mean ± SD) (*n* = 3)).

Copper oxide nanoparticles are widely used in various products and their potential toxicities in aquatic organisms and in the environment is a matter of increasing concern. In our study acute toxicity tests were performed with engineered and plant-mediated copper nanoparticles using the same conditions of experiments for both types of nanoparticles. Different doses of nanoparticles were applied to assess the toxicity of nanoparticles and the % mortality was calculated with results shown in Figure 10. Effective concentration (48 h) was determined and expressed as EC_50_, with details given in Table 2. The EC_50_ value by the definition is the concentration causing a designated sublethal effect on 50% of the test organisms within a specific exposure period [41]. Engineered CuO NPs showed a much higher toxicity with an EC_50_ value of 0.102 mg/L (with a 95% CI of 0.09–0.13 mg/L) as compared to plant copper nanoparticles (0.64 mg/L, with a 95% CI of 0.38–0.9 mg/L). In the present study, engineered and plant CuO NPs were found to have an EC_50_ level of 0.102 mg/L and 0.69 mg/L, respectively, which was more than five times lower than that of engineered CuO nanoparticles. It is, thus, obvious that *D. magna* is much more vulnerable to dispersions of engineered CuO NPs than to dispersions of engineered CuO NPs. 

Previous studies showed that CuO nanoparticles are toxic to *Daphnia*. Acute and chronic toxicity of CuO nanoparticles to *D. magna* have been investigated at different exposure conditions in recent years [42,43]. In present study, plant-mediated CuO NPs were found to have EC_50_ 0.69 ± 0.226 mg/L, which was greater than the engineered CuO NPs (0.102 ± 0.019 mg/L). EC_50_ values was found to be 0.064 mg/L by Tavares et al. [42] and 0.093 mg/L against *Daphnia* by Xiao et al. [44]. Moreover it was found that CuO nanoparticles dissolute and at higher concentration above 0.1–1 mg/L CuO nanoparticles accumulate and cause toxicity in *Daphnia* [44]. Karlsson et al. [28] explained the toxicity of CuO nanoparticles in terms of release of Cu ions. Soluble copper ions from nanoparticles increase oxidative stress in organisms and cause toxicity to the cells. Heinlaan et al. [45] also reported that the dissolved Cu were responsible to toxicity of CuO in *Daphnia* and determined the damaging effect of CuO in the midgut of *Daphnia*. In another study Jo et al. [46] evaluated the effect of preparation methods of nanoparticles suspensions on the toxicity to *Daphnia*. According to this, different preparation methods (filtration, dispersion, initial concentration and particle size) lead to differences in dissolved concentrations, resulting in the difference in the acute toxicity. It was observed that the medium of CuO nanoparticles exposed after filtration with 0.05–0.45 μm filter caused higher acute toxicity to daphnids than the unfiltered medium.

#### 3.3.2. Nanoparticles Accumulation in *Daphnia*

**Figure 11:**
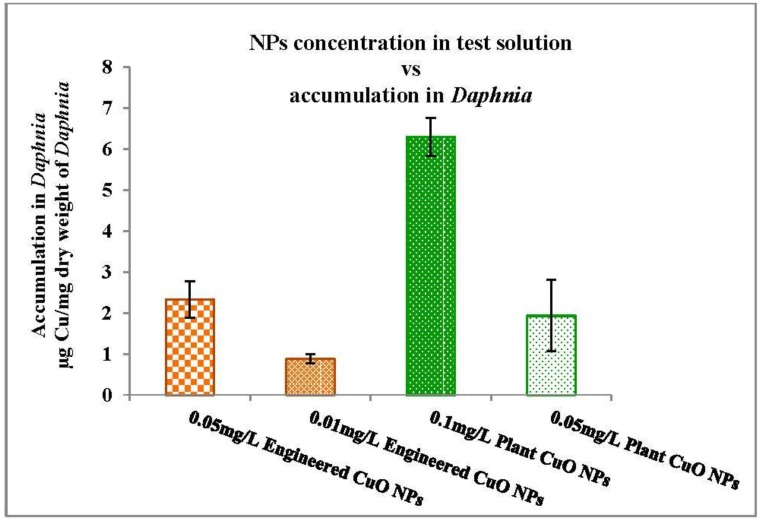
Accumulation of CuO NPs in *Daphnia*.

The accumulation profiles of engineered and plant CuO NPs are shown in Figure 11. For engineered CuO NPs at a concentration of 0.05 mg/L and 0.01 mg/L in media, the overall accumulation of Cu was 2.33 ± 0.45 μg/mg dry weight of *Daphnia* and 0.88 ± 0.01 μg/mg dry weight of *Daphnia*, respectively. For the plant CuO NP test solution at a concentration 0.1 mg/L, the accumulation was 6.3 μg/mg dry weight of *Daphnia*, and for 0.05 mg/L, test solution the accumulation was 1.933 μg/mg dry weight. The overall accumulation of engineered CuO NPs was significantly higher than the accumulation of the plant CuO NPs in *Daphnia*. But accumulation may not be due to absorption of or surface adsorption of particles on the outside of the *Daphnia*.

The relative contribution of nanoparticles and released ions to accumulation or toxicity is given in Table 3. It is expected that the toxicity is due to copper ions release from the nanoparticles, but it was unclear from the study whether toxicity can be attributed to released ions or to the nanoparticle accumulation. The percentage (%) contribution of ions from engineered CuO nanoparticles (26.0 ± 2) is higher than plant-synthesized nanoparticles (17.3 ± 1.02) at 0.05 mg/L in test solution. The dissolution or ion release mechanism of engineered and plant CuO NPs was different under the same exposure conditions.

The results of the present study indicate that ion release from the nanoparticle was more than that of the plant-mediated CuO nanoparticles. From the results it can be assumed that engineered nanoparticles were more toxic due to dissolution of copper ions from nanoparticles. This toxicity behavior has already been explained in a few studies, i.e., the soluble copper ions from CuO nanoparticles or dissolved fractions of nanoparticles largely contribute to acute toxicity in *Daphnia*. The relative contribution of particles and ions to accumulation in *Daphnia* was determined in our study at a concentration of 0.05 mg/L in test solution. Twenty-six percent Cu ions from engineered CuO nanoparticles contributed to accumulation in *Daphnia*, compared with 17.3% Cu ions from plant-synthesised. The overall accumulation of Cu for engineered CuO NPs at a concentration of 0.05 was 2.33 ± 0.45 μg/mg dry weight of *Daphnia* and for plant-synthesized CuO NPs was 1.933 μg/mg dry weight. Hence it is considered that the toxicity might be due to Cu ion or particle accumulation in *Daphnia* body. A further aspect that should be considered is the size of particles. The size of plant-synthesized nanoparticles was found to be in range of 0.1–0.25 µm, whereas that of the engineered nanoparticles was <50 nm. The nano CuO dissolute more rapidly than the bulk CuO [47]. Heinlaan et al. [23] reported the eco-toxic effect of CuO in relation to the size of particles, where nano CuO showed high toxicity than the bulk CuO. The bulk particles solubilize less and are less bioavailable to *Daphnia*. Likewise, Adam et al. [43] reported that the ionic form of Cu is more toxic, after determining the toxicity of nano CuO and CuCl_2_·H_2_O, since the dissolution was related to the toxicity. Copper salt dissolved rapidly and caused high toxicity in *Daphnia*. However, the smaller aggregates of CuO NPs dissolve more rapidly. It might be possible that the size of the nanoparticle is the reason for the dissolution of engineered CuO NPs more than the plant-synthesized CuO NPs. Plant are able to produce stable nanoparticles, phenolic compounds such as flavonoids or tannins act as stabilizing and coating agents [1,48]. This might be the other reason for the lower dissolution of plant-synthesized CuO NPs as compared to engineered CuO NPs.

The literature shows that the toxicity of nanoparticles depends on different factors and nanoparticles behave differently with different microorganisms, so it is hard to draw any conclusion about underlying mechanism of CuO nanoparticles toxicity. If the concern is green synthesis of metallic nanoparticles, the authors Markova et al. [49] and Rani and Rajasekharreddy [50] evaluated the eco-toxicological impacts of plant-mediated metallic nanoparticles against different aquatic species. The study revealed that plant-mediated nanoparticles had no significant/less toxic effect on aquatic species. The results of our study also indicate that plant-mediated CuO nanoparticles are less toxic than the engineered CuO nanoparticles. However, this is the first study that describes the eco-toxicological impacts of plant-synthesized CuO nanoparticles, so NPs toxicity should be investigated further with more carefully.

## 4. Conclusions

Synthesis of nanoparticles can be performed using a number of routinely used chemicals and physical methods. In this study, synthesis of copper oxide nanoparticles was conducted using an environmentally friendly mechanism in which aqueous leaf extract of *P. acerifolium* was utilized with copper salt. The plant-synthesized CuO nanoparticles were found to be more stable with less ion release compared to the engineered CuO NPs. In addition, hydrodynamic size and aggregation increased with the passage of time, but plant-synthesized CuO NPs fell behind in size and aggregation increased when compared to the engineered NPs. The engineered CuO NPs were more soluble than plant-mediated CuO NPs and the rate of ion release was higher than that of plant nanoparticles. Thus, plant-synthesized CuO nanoparticles were found to be more stable when compared with engineered nanoparticles. Acute nanoparticle toxicity tests proved that plant-synthesized CuO NPs are less toxic than engineered CuO NPs, which provides a new way to synthesize more environmentally friendly nanoparticles for various applications. However, it remains unclear whether both plant and engineered CuO toxicity is due to ion dissolution or if it comes from the CuO NPs themselves and their response to the environment. Further research is needed to determine the cause of copper oxide nanoparticle toxicity in order to combat potential hazards to the environment.

## Figures and Tables

**Table 1:** Zeta potential of engineered and plant-synthesized CuO NPs.

Nanoparticle Type	Time			
	Initial	24 h	48 h	72 h
Engineered CuO NPs	−11.7 ± 2.52	−2.10 ± 0.67	11.43 ± 1.62	18.13 ± 0.60
Plant CuO NPs	−9.27 ± 1.10	−3.76 ± 0.86	7.69 ± 0.35	16.25 ± 0.36

**Table 2:** EC_50_ acute toxicity of engineered and plant CuO NPs.

Test Material	EC_50_ (mg/L)	CI 95%
Engineered CuO NPs	0.102 ± 0.019	0.09–0.13
Plant CuO NPs	0.69 ± 0.226	0.38–0.9

Mean ± SD (*n* = 3), confidence of interval 95%.

**Table 3:** Relative contribution of nanoparticles and released ions to accumulation in *Daphnia*.

Actual Concentration	% Relative Contribution of Released Cu^+^ Ions to Accumulation	
	NPs_(particle)_	NPs_(ion)_
0.05 mg/L Engineered CuO NPs	74.0 ± 2.0	26.0 ± 2
0.01 mg/L Engineered CuO NPs	83.7 ± 2.3	16.3 ± 2.3
0.1 mg/L Plant CuO NPs	71.94 ± 3.30	28.06 ± 3.30
0.05 mg/L Plant CuO NPs	82.87 ± 1.02	17.3 ± 1.02

Mean ± SD (*n* = 3).
